# Are common mental disorders more persistent over time? Insights from the Netherlands Mental Health Survey and Incident Studies (NEMESIS)

**DOI:** 10.1017/S0033291726105467

**Published:** 2026-09-03

**Authors:** Artemis Gkitakou, Margreet Ten Have, Neeltje M. Batelaan, Annemarie I. Luik, Brenda W.J.H. Penninx

**Affiliations:** 1 Department of Psychiatry, Amsterdam UMC, Vrije Universiteit Amsterdam, Public Health Research Institute, Amsterdam, Netherlands; 2 Trimbos Institute, Netherlands Institute of Mental Health and Addiction, Utrecht, Netherlands

**Keywords:** anxiety disorder, cohort, epidemiology, mental health, mood disorder, persistence, substance use disorder, trends

## Abstract

**Background:**

Recent increases in common mental disorder prevalence could partly reflect greater persistence of existing disorders. We examined 3-year persistence for anxiety, mood, and substance use disorders, defined as meeting diagnostic criteria at baseline and again during 3-year follow-up. We compared persistence rates with those observed 12 years earlier and investigated (changes in) determinants of persistence.

**Methods:**

Data were from the third Netherlands Mental Health Survey and Incidence Study (NEMESIS-3), a population-based study (*N* = 6,194; 18–75 years; interviewed 2019–2022 and 2023–2024). A slightly modified Composite International Diagnostic Interview 3.0 assessed DSM-5 and DSM-IV diagnoses. Persistence was described from two perspectives: narrow (presence of a mental disorder of the same diagnostic category during follow-up) and broad (presence of any anxiety, mood, and/or substance use disorder during follow-up). Twelve-year trends were examined by comparing DSM-IV persistence in NEMESIS-3 with NEMESIS-2 (*N* = 6,646; interviewed 2007–2009 and 2010–2012) among adults aged 18–64 years.

**Results:**

In NEMESIS-3, 3-year narrow persistence was 41.6% for anxiety, 48.0% for mood, and 40.3% for substance use disorders. Broad persistence was higher: 57.1%, 63.2%, and 55.1%, respectively. Persistence increased in 12 years across all disorder groups: broad persistence for any mental disorder was 42.7% in NEMESIS-2 and 57.9% in NEMESIS-3. Higher broad persistence was significantly associated with younger age, living in a city, childhood trauma, and near-daily drug use in multivariable analyses. These associations were stable over time.

**Conclusions:**

Three-year persistence of common mental disorders is substantial and has increased compared with 12 years earlier, suggesting growing challenges for treatment and long-term care.

## Introduction

Common mental disorders, such as anxiety, mood, and substance use disorders, are among the leading contributors to the global disease burden (Vigo, Thornicroft, & Atun, 2016). Despite growing awareness of mental health issues and improved access to care (Thornicroft et al., 201 7), evidence suggests that the 12-month prevalence of these disorders has increased over the last decade (Gkitakou et al., 2026; Goodwin et al., 2022; Morris, Hill, Brugha, & McManus, 2025; Slade et al., 2024; Ten Have et al., 2023; Yu et al., 20 20). Specifically, the 12-month prevalence of any anxiety disorder increased from 10.1% to 15.6%, any mood disorder from 6.0% to 10.8%, and substance use disorders from 5.5% to 7.1% between 2007–2009 and 2019–2022 (Ten Have et al., 2023). However, these results are not entirely consistent, as some studies reported declines in anxiety and depressive symptoms (Beller et al., 202 1; Martinsen et al., 2026), or in substance use disorder prevalence (Slade et al., 2024), and older studies reported stable trends in depressive disorders (Bretschneider et al., 2018; Dobson, Vigod, Mustard, & Smith, 2020; Markkula et al., 2017). Recent increases in anxiety and mood disorders prevalence could not be explained by changes in common sociodemographic or environmental risk factors (Gkitakou et al., 202 6). This increased prevalence may reflect a rising incidence (Tuithof et al., 2026), but could also indicate greater persistence over time. Understanding trends in the long-term course of these disorders is therefore essential to guide prevention, intervention, and recovery-focused care. Longitudinal research consistently shows that common mental disorders often follow a recurrent and/or persistent course (Penninx et al., 2011; Scholten et al., 2021; Ten Have et al., 2022a; Verduijn et al., 2017). In evaluating persistence, some studies adopt a narrow perspective, considering only the persistence of the same diagnostic disorder at follow-up. Others use a broader perspective, for example, capturing the presence of any mental disorder at follow-up, regardless of diagnostic category (Penninx et al., 2011; Ten Have et al., 2022a). The broad perspective is particularly important because it reflects the ongoing burden of illness even when diagnoses shift over time (Scholten et al., 2016; Ten Have et al., 2026).

Cohort studies such as the Netherlands Study of Depression and Anxiety (NESDA), the Netherlands Mental Health Survey and Incidence Study (NEMESIS), and the US National Epidemiologic Survey on Alcohol and Related Conditions (NESARC), all indicate that a substantial proportion of individuals with anxiety or depressive disorders continue to meet diagnostic criteria years later (Mackenzie, El-Gabalawy, Chou, & Sareen, 2014; Penninx et al., 2011; Scholten et al., 2021; Ten Have et al., 2022a; Verduijn et al., 2017). NESDA primarily includes adult clinical patients, whereas NEMESIS and NESARC are based on the general population. In NESDA, 30.6% of individuals with pure anxiety disorder and 28.8% of those with pure depressive disorder continued to have a disorder from the same diagnostic category after 2 years, which was even higher (39.6%) among those with comorbid anxiety–depression (Penninx et al., 2011). Quite similarly, in NEMESIS-2, 20.5% of individuals with any anxiety disorder and 26.0% of individuals with major depressive disorder (MDD) at baseline still met diagnostic criteria for any anxiety disorder or MDD, respectively, over a 3-year follow-up (Ten Have et al., 2022a). Comparable findings were reported in NESARC using a slightly different definition of persistence (endorsing a past-year disorder at both baseline and follow-up): 29.5% of individuals with any anxiety disorder and 31.7% of those with any mood disorder continued to have the same diagnostic category after 3 years (Mackenzie et al., 201 4). Notably, when applying a broader definition of persistence, rates were substantially higher. For instance, in NEMESIS-2, 38.2% of those with any anxiety disorder and 38.9% of those with MDD met criteria for any mental disorder (anxiety, mood, or substance use disorder) after 3 years (Ten Have et al., 2022a).

Substance use disorders can also follow a rather persistent course. Data from NESARC indicate that 30.9% of individuals with a drug use disorder at baseline met diagnostic criteria for drug use disorder again during the 3-year follow-up (Fenton et al., 2011). NEMESIS-2 reported that 29.5% of individuals with 12-month alcohol use disorder at baseline still met diagnostic criteria at 3-year follow-up assessment (Tuithof et al., 2013). Definitions of persistence vary across longitudinal studies, with some applying a narrow definition and others a broad one. Notably, no study has applied a broad definition to the course of substance use disorder. Moreover, although sociodemographic determinants such as age, sex, education, and living situation have been examined in relation to persistence of common mental disorders, findings have been inconsistent (Mackenzie et al., 201 4; Penninx et al., 2011; Ten Have et al., 2022a) and evidence on changes in the strength of these associations over time is lacking. Most importantly, it is unknown whether persistence itself has changed over time, as this could contribute to increasing prevalence trends (Gkitakou et al., 202 6; Ten Have et al., 2023; Yu et al., 20 20). Using 3-year longitudinal data from two large, representative adult cohorts (NEMESIS-2, baseline 2007–2009; NEMESIS-3, baseline 2019–2022), this study examines and compares for the first time: (1) narrow and broad persistence rates of anxiety, mood, and substance use disorder within a single study, (2) trends in 12 years of persistence outcomes, and (3) sociodemographic determinants of persistence, including sex, age, living situation, employment status, education level, urbanicity, income, negative life event, childhood trauma, smoking, physical activity, chronic physical conditions, alcohol and drug use frequency, and whether their associations have changed over time.

## Methods

### Study design

NEMESIS-2 and NEMESIS-3 (the second and third Netherlands Mental Health Survey and Incidence Studies) used a stratified, multistage random sampling design to obtain a representative sample of the Dutch general population. Municipalities were selected at random, with the four largest cities (Amsterdam, Rotterdam, The Hague, and Utrecht) preincluded (De Graaf, Ten Have, & Van Dorsselaer, 2010; Ten Have et al., 2022b, 2023). In NEMESIS-3, a random sample of individuals aged 18–75 years was drawn from the national population register (Basisregistratie Personen) (Ten Have et al., 2022b). In NEMESIS-2, private household addresses were drawn from postal registers, and within each household, one individual aged 18–64 years was selected using the most recent birthday method (De Graaf et al., 2010). Individuals who were institutionalized (except temporarily) or not fluent in Dutch were excluded in both studies. In both studies, participants received a small financial compensation for their participation at each wave. In NEMESIS-3, ethical approval was exempt from approval under the Dutch Medical Research Involving Human Subjects Act (WMO), whereas for NEMESIS-2 approval was obtained due to saliva collection. Comprehensive methodological details are available in previous publications (De Graaf et al., 2010; Ten Have et al., 2022b). Written informed consent was obtained from all participants at each wave.

### Fieldwork and follow-up

In NEMESIS-3, baseline interviews were conducted from November 2019 to March 2022 with a response rate of 54.6% (*n* = 6,194). After excluding 59 deceased participants, 6,135 respondents were approached for follow-up ~3 years later (January 2023–November 2024), of whom 4,688 (76.4%) completed the interview (mean interval 2 years and 310 days; mean interview duration: 76 minutes). Attrition at follow-up was significantly associated with having a mental disorder in the 12 months before baseline (Ten Have et al., 2025). In NEMESIS-2, baseline interviews were conducted from November 2007 to July 2009 with a response rate of 65.1% (*n* = 6,646). Of the 6,596 participants still alive, 5,303 (80.4%) completed follow-up ~3 years later (November 2010–July 2012; mean interval 3 years and 7 days; mean interview duration: 84 minutes). No significant associations were observed between having a mental disorder in the 12 months before baseline and attrition at follow-up in NEMESIS-2 after controlling for sociodemographics (De Graaf, Van Dorsselaer, Tuithof, & Ten Have, 2013).

Both studies closely represented the Dutch population at baseline, although younger individuals, those with lower or higher secondary education, and those living without a partner were slightly underrepresented (De Graaf et al., 2013; Ten Have et al., 2025). To enhance generalizability, post-stratification weights were applied in each study, adjusting for selective nonresponse at baseline and attrition at follow-up. The weighting factors for both studies included sex, age, partner status, education level, and urbanicity based on Statistics Netherlands data (2008 for NEMESIS-2, 2020 for NEMESIS-3). The weighting factor for NEMESIS-3 additionally corrected for any 12-month mental disorder at baseline, as psychopathology was a predictor of attrition in this study but not in NEMESIS-2. After weighting, the sociodemographic characteristics of follow-up participants closely matched those of the Dutch population at baseline (De Graaf et al., 2013; Ten Have et al., 2022b, 2025).

### Diagnostic assessments

Mental disorders were assessed using the Composite International Diagnostic Interview (CIDI) 3.0, a fully structured, lay-administered diagnostic interview developed for the World Mental Health Survey Initiative (Kessler & Üstün, 2004). This instrument has demonstrated reliable validity for assessing common mental disorders according to DSM-IV (Haro et al., 2006). NEMESIS-2 used the standard CIDI 3.0, while NEMESIS-3 applied a slightly modified version to be able to assess DSM-5 disorders, alongside DSM-IV disorders (Ten Have et al., 2022b). In both studies and both waves, disorders from the following diagnostic categories were assessed: (1) anxiety disorders (including panic disorder, agoraphobia, social anxiety disorder, and generalized anxiety disorder), (2) mood disorders (including major depressive disorder, persistent depressive disorder/dysthymia, and bipolar disorder), and (3) substance use disorders (including alcohol and drug use disorders). Drug use disorder included the following substances: sedatives (sleeping pills or tranquilizers), stimulants or stimulant medications, pain relievers, cannabis, cocaine, ecstasy, GHB, ketamine, opiates (heroin), solvents, sniffing agents, or other drugs. Among persons with a drug use disorder, cannabis use disorder was the most common, followed by sedative and pain reliever use disorders, in both NEMESIS-2 (43.0%, 33.8%, and 16.6%) and NEMESIS-3 (61.5%, 18.5%, and 15.7%). All analyses were conducted at the level of the three diagnostic categories.

A pure disorder was defined as the presence of one or more disorders of a single diagnostic category (i.e. anxiety, mood, and/or substance use disorders) without the presence of disorders from the other categories. Specifically, a pure anxiety disorder was defined as the presence of one or more anxiety disorders without comorbid mood or substance use disorders. Similarly, a pure mood disorder was defined as the presence of one or more mood disorders without anxiety or substance use disorders, and a pure substance use disorder as the presence of one or more substance use disorders without anxiety or mood disorders. Comorbidity was defined as the presence of disorders from two or more diagnostic categories at baseline. So, this could be anxiety and mood disorders without substance use disorder, mood and substance use disorders without anxiety disorder, anxiety and substance use disorders without mood disorder, or disorders from all three diagnostic categories.

Persistence was defined as having a 12-month disorder at baseline and meeting diagnostic criteria again at any time during the 3-year follow-up. Two perspectives were applied: narrow persistence, defined as the presence of a disorder of the same diagnostic category during follow-up, and broad persistence, defined as the presence of any mental disorder (i.e. any anxiety, mood, and/or substance use disorder) during follow-up.

NEMESIS-3 provided updated estimates of three-year persistence of common mental disorders (i.e. anxiety, mood, and/or substance use disorders) in a recent period, while comparison with NEMESIS-2 allowed the examination of changes in persistence over time. Changes over time refer to differences in persistence between NEMESIS-2 and NEMESIS-3.

### Clinical characteristics

Health care utilization for mental problems or alcohol or drug use problems over the past 12 months was categorized into three mutually exclusive groups: none (no professional care), primary care only, and specialized mental health care.

### Determinants

Baseline determinants assessed during the interview included sex, age, living situation, employment status, education level, urbanicity, income, negative life event, childhood trauma, smoking, physical activity, chronic physical conditions, and alcohol and drug use frequency. These variables were measured comparably in both studies to allow direct comparisons (De Graaf et al., 2010; Ten Have et al., 2022b). Living situation was categorized as living with or without a partner; employment status as having a paid job or not. Education level was grouped into primary/lower secondary (low), higher secondary (middle), or higher vocational/university (high); urbanicity as rural or city. Income was based on household income, divided into three categories: lowest 25%, middle 50%, and highest 25%. Negative life event was assessed using the Brugha Life Events section, covering 10 adverse events in the past 12 months (e.g. relative’s death and divorce) (Brugha, Bebbington, Tennant, & Hurry, 1985); a binary variable indicated at least one event. Childhood trauma included emotional neglect, psychological or physical abuse on ≥2 occasions, or sexual abuse ≥1 time before age 16 years. Smoking referred to use in the past 4 weeks, and not physically active was defined as nonadherence to the Dutch healthy exercise norm (≥30 min of moderate-intensity activity on at least 5 days) (Craig et al., 2003). Chronic physical conditions were self-reported from a standardized list of 17 diseases (e.g. diabetes, arthritis, and cardiovascular disease) and coded as a binary variable (≥1 condition) if medically treated or monitored in the past 12 months (De Graaf et al., 2010). Alcohol use frequency was categorized as nondrinking, nondaily drinking (any alcohol use in the past 12 months that was less frequent than 5–7 days per week, including both irregular and regular drinking patterns), or near-daily drinking (5–7 days per week). Drug use frequency was dichotomized as near-daily use (5–7 days per week; yes/no), reflecting the highest frequency of any drug use in the past 12 months. Both frequency measures were derived from questions in the CIDI 3.0.

### Statistical analysis

General characteristics of participants with any anxiety, any mood, any substance use disorder, or any mental disorder at baseline in NEMESIS-3 (DSM-5, 18–75 years) were summarized using frequency tables. The persistence rates for these main diagnostic categories, pure disorders, and comorbid disorders in NEMESIS-3 (DSM-5, 18–75 years) were calculated using two perspectives: narrow (presence of a mental disorder of the same diagnostic category during follow-up) and broad (presence of any anxiety, mood, and/or substance use disorder during follow-up). For trend analyses comparing persistence rates between NEMESIS-2 and NEMESIS-3, DSM-IV criteria were applied, and analyses were restricted to adults aged 18–64 years to ensure consistency across studies. Persistence rates were calculated in the same manner, using narrow and broad definitions. Differences in persistence rates between studies were examined using chi-square tests (unadjusted) and logistic regression analyses adjusted for sex and age. Determinants of persistence of any mental disorder (broad perspective) were examined in the combined dataset of NEMESIS-2 and NEMESIS-3 (DSM-IV, 18–64 years). Logistic regression was used, first in separate models for each determinant (sex, age, living situation, employment status, education level, urbanicity, income, negative life event, childhood trauma, smoking, physical activity, chronic physical conditions, and alcohol and drug use frequency) and second in a multivariable model adjusting for all determinants simultaneously. To assess whether the strength of determinants differed between studies, multiplicative interactions were evaluated using univariate logistic regressions, and additive interactions were tested using univariate generalized linear models for binary outcomes, estimating risk differences. Multiplicative interactions capture deviations on a relative scale, whereas additive interactions reflect excess risk on an absolute scale (VanderWeele & Knol, 2014). Evaluating both scales offers a more comprehensive understanding of whether determinants of persistence have changed over time (Coleman et al., 2020).

All statistical analyses were conducted using Stata 16.1 (StataCorp LLC, College Station, TX, USA; https://www.stata.com) on Windows, with post-stratification weights applied to ensure population representativeness.

## Results

Table 1 presents the baseline characteristics of participants with a 12-month DSM-5 mental disorder in NEMESIS-3 (2019–2022). More than half of those with any anxiety, any mood, or any mental disorder were female, whereas only a third of those with substance use disorder were female. The age distribution was similar across those with any anxiety (41.2%) or any mood (41.0%) disorder, with the proportion of younger adults (18–34 years) being notably higher among participants with substance use disorder (57.3%).

**Table 1.** Descriptive characteristics of respondents with any 12-month anxiety, mood, substance use disorder, or any mental disorder according to DSM-5 in NEMESIS-3 at baseline (2019–2022; age 18–75 years), in unweighted numbers with weighted percentages Table 1. long description.

NEMESIS-3 (2019–2022)	Persons with any anxiety disorder	Persons with any mood disorder	Persons with any substance use disorder	Persons with any mental disorder
	*N* = 402	*N* = 371	*N* = 290	*N* = 828
	(%)	(%)	(%)	(%)
Demographic characteristics				
Female	61.8	60.5	32.9	53.4
Young adults (18–34 years)	41.2	41.0	57.3	45.7
Living without partner	52.3	53.8	59.0	53.2
No paid job	41.0	43.4	29.9	36.4
Education				
Low	26.6	27.4	21.2	24.0
Middle	42.7	44.9	37.7	42.7
High	30.6	27.7	41.1	33.4
Living in a city	64.9	67.8	66.7	64.2

### Three-year persistence in NEMESIS-3

In NEMESIS-3, the 3-year persistence of mental disorders was substantial across all diagnostic categories. Narrow persistence – defined as presence of a mental disorder of the same diagnostic category during follow-up – was 41.6% for any anxiety disorder, 48.0% for any mood disorder, and 40.3% for any substance use disorder. When applying the broad perspective, which considers the presence of any mental disorder during follow-up, persistence was higher: 57.1%, 63.2%, and 55.1% for any anxiety, mood, and substance use disorder, respectively. Overall, broad persistence of any mental disorder reached 55.2%. A similar pattern, with higher rates when switching from a narrow to a broad perspective, was observed among individuals with pure disorders. Persistence rates were particularly high among those with comorbid disorders when using a broad perspective, varying between 62.5% and 75.8% (Table 2).

**Table 2.** Three-year persistence rates of DSM-5 mental disorders among respondents with any 12-month disorder at baseline in NEMESIS-3 (2019–2024; age 18–75 years), in unweighted numbers with weighted percentages Table 2. long description.

NEMESIS-3	*N* at T0	Presence of a mental disorder of the same diagnostic category during T0–T1 (narrow perspective)	Presence of any mental disorder^a^ during T0–T1 (broad perspective)
DSM–5 disorder at T0		% (95% CI)	% (95% CI)
Any mental disorder^a^	828		55.2 (51.1, 59.1)
Disorder categories			
Any anxiety disorder^b^	402	41.6 (35.9, 47.6)	57.1 (52.0, 62.1)
Any mood disorder^b^	371	48.0 (41.8, 54.2)	63.2 (55.7, 70.2)
Any substance use disorder^b^	290	40.3 (34.5, 46.4)	55.1 (49.2, 60.9)
Pure disorders			
Pure anxiety disorder^c^	224	17.1 (12.3, 23.1)	43.5 (37.9, 49.3)
Pure mood disorder^c^	192	24.8 (18.7, 32.2)	53.4 (45.4, 61.2)
Pure substance use disorder^c^	206	32.4 (25.3, 40.5)	50.9 (43.5, 58.3)
Comorbid disorder			
Anxiety and mood disorder, no substance use disorder	122	66.1^d^ (56.3, 74.8)	75.8 (68.2, 82.1)
Anxiety and substance use disorder, no mood disorder	27	34.0^d^ (20.2, 51.3)	63.5 (42.2, 80.6)
Mood and substance use disorder, no anxiety disorder	28	41.3^d^ (25.6, 58.9)	67.0 (44.8, 83.5)
All three disorders	29	62.5^d^ (34.9, 83.9)	62.5 (34.9, 83.9)

*Note:* Narrow persistence refers to the presence of a disorder of the same diagnostic category during follow-up. The three diagnostic categories were: (1) anxiety disorders (panic disorder, agoraphobia, social anxiety disorder, and generalized anxiety disorder), (2) mood disorders (major depressive disorder, persistent depressive disorder, and bipolar disorder), and (3) substance use disorders (alcohol and drug use disorders).

a Any mental disorder refers to any DSM-5 anxiety, mood, and/or substance use disorder.

b Includes those also meeting criteria for another mental disorder.

c Excluding those who also meet criteria for another mental disorder (i.e. anxiety, mood, and/or substance use disorder).

d For the narrow perspective, a mental disorder of the same diagnostic category was here defined as having a disorder from either one or both of the diagnostic categories. For example, participants with comorbid anxiety and mood disorders but no substance use disorder at baseline were considered persistent if anxiety and/or mood disorders, but not substance use disorder, were present at follow-up.

### Trends in 3-year persistence

Three-year broad persistence increased substantially across all disorders compared with 12 years earlier, with any mental disorder rising from 42.7% in NEMESIS-2 to 57.9% in NEMESIS-3. When using the narrow perspective, persistence of any anxiety disorder increased significantly from 24.4% in NEMESIS-2 to 40.1% in NEMESIS-3; similarly, any mood disorder increased from 33.5% to 48.8% and substance use disorder from 33.7% to 45.5%. When using the broad perspective, the increase was comparable, with any anxiety disorder rising from 48.5% in NEMESIS-2 to 58.8% in NEMESIS-3, any mood disorder from 49.6% to 64.7%, and substance use disorder from 48.7% to 60.5%. These differences were confirmed across both perspectives by logistic regression analyses adjusted for sex and age, with significant odds ratios (ORs) across all disorders except for substance use disorder, where the change was no longer statistically significant after adjustment (Table 3). Findings indicating higher persistence in more recent years were, although not always significantly and consistently in the small subgroups, observed across different types of pure and comorbid disorders (Table 3).

**Table 3.** Trends in 3-year persistence rates of DSM-IV mental disorders among respondents with any 12-month disorder at baseline in NEMESIS-2 (2007–2012) and NEMESIS-3 (2019–2024) among adults aged 18–64 years: comparison by disorder type and number of disorders, in unweighted numbers with weighted percentages, unadjusted *p*-values, and weighted odds ratios adjusted for age and sex Table 3. long description.

DSM-IV disorder at T0	*N* at T0		Presence of a mental disorder of the same diagnostic category during T0–T1 (narrow perspective)		*p*-value NEMESIS-2 vs. NEMESIS-3	Sex- and age-adjusted	Presence of any mental disorder^a^ during T0–T1 (broad perspective)		*p*-value NEMESIS-2 vs. NEMESIS-3	Sex- and age-adjusted
	NEMESIS-2	NEMESIS-3	NEMESIS-2 % (95% CI)	NEMESIS-3% (95% CI)		aOR (95% CI)	NEMESIS-2% (95% CI)	NEMESIS-3% (95% CI)		aOR (95% CI)
Any mental disorder^a^	703	691					42.7 (37.8, 47. 7)	57.9 (53.6, 62.2)	<0.01	1.81 (1.39, 2.36)
Disorder categories										
Any anxiety disorder^b^	324	318	24.4 (17.2, 33.5)	40.1 (35.0, 45.5)	<0.01	2.07 (1.25, 3.44)	48.5 (41.6, 55.5)	58.8 (53.9, 63.5)	0.02	1.51 (1.07, 2.13)
Any mood disorder^b^	323	332	33.5 (27.2, 40.5)	48.8 (42.2, 55.5)	<0.01	1.91 (1.25, 2.93)	49.6 (43.1, 56.1)	64.7 (57.3, 71.4)	<0.01	1.86 (1.24, 2.80)
Any substance use disorder^b^	225	219	33.7 (26.1, 42.3)	45.5 (37.9, 53.4)	0.04	1.57 (0.98, 2.53)	48.7 (40.6, 56.8)	60.5 (52.1, 68.3)	0.05	1.56 (0.98, 2.51)
Pure disorders										
Pure anxiety disorder^c^	195	183	8.0 (4.5, 13.9)	18.3 (13.0, 25.3)	0.02	2.59 (1.18, 5.70)	34.7 (27.1, 43.2)	46.8 (41.6, 52.1)	0.02	1.63 (1.07, 2.48)
Pure mood disorder^c^	193	186	13.7 (8.8, 20.8)	26.0 (20.3, 32.5)	<0.01	2.19 (1.10, 4.35)	33.3 (26.7, 40.7)	56.6 (48.5, 64.4)	<0.01	2.51 (1.53, 4.12)
Pure substance use disorder^c^	169	160	17.5 (11.3, 26.2)	36.5 (27.1, 47.1)	<0.01	2.57 (1.29, 5.13)	37.4 (28.1, 47.7)	54.5 (44.1, 64.6)	0.02	1.88 (1.02, 3.47)
Comorbid disorder										
Anxiety and mood, no substance use disorder	90	103	52.7* (37.7, 67.3)	63.1* (53.3, 71.8)	0.25	1.58 (0.77, 3.24)	60.3 (46.6, 72.5)	72.5 (62.4, 80.6)	0.11	1.83 (0.93, 3.58)
Anxiety and substance use, no mood disorder	16	16	64.2* (45.5, 79.5)	44.8* (22.7, 69.2)	0.29	0.51 (0.09, 2.96)	67.9 (49.3, 82.2)	69.1 (41.5, 87.6)	0.95	1.05 (0.16, 7.16)
Mood and substance use, no anxiety disorder	17	27	57.8* (30.4, 81.1)	47.3* (28.8, 66.7)	0.59	0.63 (0.14, 2.84)	78.9 (52.3, 92.7)	76.7 (54.5, 90.1)	0.88	0.91 (0.16, 5.02)
All three disorders	23	16	88.6* (69.6, 96.4)	80.6* (51.5, 94.2)	0.53	0.71 (0.07, 7.27)	88.6 (69.6, 96.4)	80.6 (51.5, 94.2)	0.53	0.71 (0.07, 7.27)

*Note*: Narrow persistence refers to the presence of a disorder of the same diagnostic category during follow-up. The three diagnostic categories were: (1) anxiety disorders (panic disorder, agoraphobia, social anxiety disorder, and generalized anxiety disorder), (2) mood disorders (major depressive disorder, dysthymia, and bipolar disorder), and (3) substance use disorders (alcohol and drug use disorders).

Adjusted model: adjusted for sex and age.

a Any mental disorder refers to any DSM-IV anxiety, mood, and/or substance use disorder.

b Those also meeting criteria for another mental disorder.

c Excluding those who also meet criteria for another mental disorder (i.e. anxiety, mood, and/or substance use disorder).

* For the narrow perspective, a mental disorder of the same diagnostic category was here defined as having a disorder from either one or both of the diagnostic categories. For example, participants with comorbid anxiety and mood disorders but no substance use disorder at baseline were considered persistent if anxiety and/or mood disorders, but not substance use disorder, were present at follow-up.

### Clinical characteristics

Mental health care utilization among individuals with any anxiety, mood, and/or substance use disorder did not differ significantly between the studies. Specifically, primary care use only was 12.5% in NEMESIS-2 and 16.5% in NEMESIS-3, and specialized care use was 26.3% and 26.2%, respectively.

### Determinants of persistence

The determinants of 3-year persistence of any mental disorder at baseline using the broad perspective were examined in the combined dataset of NEMESIS-2 and NEMESIS-3. In unadjusted models, younger age, living without a partner, no paid job, living in a city, low and middle income, childhood trauma, smoking, and near-daily drug use were associated with higher persistence. In the fully adjusted model (including all determinants), younger age (OR = 1.88, 95% CI: 1.35–2.61), living in a city (OR = 1.40, 95% CI: 1.05–1.87), childhood trauma (OR = 1.48, 95% CI: 1.13–1.94), and near-daily drug use (OR = 1.71, 95% CI: 1.15–2.56) remained significant predictors of higher persistence (Table 4).To further assess whether the strength of these determinants changed between NEMESIS-2 and NEMESIS-3, we examined their interactions with study (NEMESIS-2 vs. NEMESIS-3). No significant interactions were observed (Supplementary Table S1).

**Table 4.** Determinants of 3-year persistence of any DSM-IV mental disorder (broad definition) among respondents with any 12-month disorder at baseline in the combined dataset of NEMESIS-2 and NEMESIS-3 (18–64 years), in weighted odds ratios (OR) Table 4. long description.

Any mental disorder (broad perspective)	Unadjusted model	Adjusted model
	OR (95% CI)	aOR (95% CI)
Determinants		
Female	1.05 (0.82, 1.35)	1.04 (0.80, 1.35)
Young adult (18–34 years)	**1.92 (1.53, 2.42)**	**1.88 (1.35, 2.61)**
Living without partner	**1.54 (1.22, 1.93)**	1.16 (0.86, 1.57)
No paid job	**1.34 (1.04, 1.73)**	1.03 (0.75, 1.41)
Education (ref = high)		
Low	1.14 (0.86, 1.51)	0.90 (0.63, 1.27)
Middle	1.04 (0.79, 1.37)	1.00 (0.73, 1.37)
Living in a city	**1.35 (1.03, 1.76)**	**1.40 (1.05, 1.87)**
Income (ref = high)		
Low	**1.82 (1.27, 2.61)**	1.37 (0.88, 2.14)
Middle	**1.49 (1.07, 2.08)**	1.26 (0.86, 1.84)
Negative life event	1.13 (0.85, 1.51)	1.06 (0.78, 1.44)
Childhood trauma	**1.44 (1.11, 1.86)**	**1.48 (1.13, 1.94)**
Smoking	**1.40 (1.09, 1.81)**	1.22 (0.91, 1.64)
Not physically active	1.14 (0.91, 1.44)	1.11 (0.86, 1.43)
≥1 chronic physical condition	0.93 (0.75, 1.14)	0.92 (0.69, 1.22)
Alcohol use frequency (ref = non-drinking)		
Non-daily drinking	0.78 (0.57, 1.08)	0.75 (0.49, 1.13)
Near-daily drinking	0.72 (0.47, 1.09)	0.85 (0.52, 1.39)
Near-daily drug use	**1.73 (1.22, 2.43)**	**1.71 (1.15, 2.56)**

*Note:* Data were weighted using post-stratification to ensure generalization to Dutch population.

OR, odds ratio; aOR, adjusted odds ratio; CI, confidence intervals; ref, reference.

Statistically significant (*p*-value<0.05) differences in odds ratios (ORs) for unadjusted and adjusted models are indicated in **bold**.

aOR was adjusted for all other variables in this table.

## Discussion

In NEMESIS-3, 3-year persistence of common mental disorders (anxiety, mood, and/or substance use disorders) was comparable and high across all disorders, particularly when persistence was defined broadly (i.e. considering the persistence of any mental (anxiety, mood, and/or substance use) disorder during follow-up). For instance, in NEMESIS-3, of the 828 persons aged 18–75 years with any DSM-5 mental disorder in the 12 months before baseline, 55.2% had any mental disorder somewhere during the subsequent 3-year follow-up. Persistence was higher in the most recent years (2019–2024) than 12 years earlier (2007–2012), with broad persistence rates among persons aged 18–64 years with any DSM-IV mental disorder increasing from 42.7% to 57.9%. In the combined dataset of adults aged 18–64 years, younger age, living without a partner, no paid job, living in a city, low and middle income, childhood trauma, smoking, and near-daily drug use were associated with higher persistence of any DSM-IV mental disorder in univariate analyses. Of these, younger age, living in a city, childhood trauma, and near-daily drug use remained significantly associated with persistence in multivariate analyses including all determinants.

Our results confirm the importance of considering persistence from a broad perspective, consistent with earlier work (Penninx et al., 2011; Solis et al., 2021; Ten Have et al., 2022a). It emphasizes the dynamic nature of common mental disorders, in which individuals often do not fully recover but instead transition from one disorder to another (Scholten et al., 2016; Ten Have et al., 2026). From a narrow perspective, a mental disorder of the same diagnostic category may appear to remit; however, a broad perspective captures ongoing or newly arisen psychopathology and thus more accurately reflects the general continuity of mental illness. For example, NESDA showed substantial diagnostic transitions, with 31% of individuals with an anxiety disorder and 12.1% of those with a depressive disorder developing a new anxiety or depressive disorder within 4 years (Scholten et al., 2016). Also, NEMESIS showed that individuals with a pure depressive disorder may develop an anxiety disorder at subsequent waves (Ten Have et al., 2026). Higher persistence under the broad definition may also reflect the high rates of comorbidity among anxiety, mood, and substance use disorders (Lai, Cleary, Sitharthan, & Hunt, 2015; Saha et al., 2020). Many individuals meet criteria for multiple disorder categories simultaneously, and such comorbidity often persists or evolves over time rather than fully resolving (Penninx et al., 2011; Solis et al., 2021; Ten Have et al., 2022a; Verduijn et al., 2017). This pattern is evident in our results as well: the comorbid groups exhibited the highest persistence rates compared with the other disorder groups defined in our study.

When comparing 3-year persistence of common mental disorders between NEMESIS-2 (2007–2012) and NEMESIS-3 (2019–2024), we observed a substantial increase across all disorders. While the persistence of common mental disorders has long been recognized as a major concern (Penninx et al., 2011; Scholten et al., 2021; Ten Have et al., 2022a; Verduijn et al., 2017), our findings provide clear evidence that persistence is now higher than it was 12 years ago. The higher persistence over time may partly reflect greater awareness of mental health issues and corresponding changes in symptom reporting. In recent years, improved awareness, reduced stigma, and more systematic assessment of mental health (Pescosolido, Halpern-Manners, Luo, & Perry, 2021) may have enabled individuals to better recognize mental health problems, which could contribute to increased reporting. However, we believe that these factors account for only a limited part of the observed increase in disorder persistence, as the clinical assessment instrument used measures symptoms rather than self-identified disorders, and is therefore less influenced by shame or stigma. As similar methodology was used in NEMESIS-2 and NEMESIS-3, this cannot explain different persistence rates either. The increase in persistence rates coincides with broader deteriorations in the social environment, including climate change and armed conflict (Emergency Events Database (EM-DAT), 2025; United Nations High Commissioner for Refugees (UNHCR), 2024). Although such deteriorations have been shown to be linked to poorer mental health outcomes (Charlson et al., 2019; Clayton, 2021), our observational data cannot infer such causality.

Interestingly, it is not persistence alone that has shown an increasing trend in the last decade. NEMESIS studies also show rising incidence (Tuithof et al., 2026) and mental health-care use (Nuyen et al., 2025). Our findings indicate that increased persistence has additionally contributed to the observed rise in prevalence (Penninx et al., 2011). Specifically, between 2007–2009 and 2019–2022, the 12-month prevalence of any DSM-IV common mental disorder rose from 17.4% to 26.1% (Ten Have et al., 2023). Over a comparable period, the 3-year first incidence of any DSM-IV common mental disorder rose from 8.5% (2007–2012) to 14.0% (2019–2024) (Tuithof et al., 2026). In addition, the proportion of the general population using specialized mental health care use increased from 6.2% (2007–2009) to 10.0% (2019–2022) (Nuyen et al., 2025). Together (Nuyen et al., 2025; Ten Have et al., 2023; Tuithof et al., 2026; Yu et al., 20 20), this suggests a consistent decline in mental health over just one decade and highlights the urgency for public health action.

When exploring mental health care utilization among individuals with any mental disorder, we found no significant differences between NEMESIS-2 and NEMESIS-3. Specifically, the proportions of individuals receiving no care, primary care only, or specialized mental health care were comparable between the studies, suggesting that differences in mental health care utilization are unlikely to explain the observed trends in persistence.

In the adjusted multivariable model of the combined dataset, only younger age, living in a city, childhood trauma, and near-daily drug use remained significant determinants of persistence. Findings from NESARC are consistent with ours, showing that younger age is associated with higher 3-year persistence of mood and substance use disorders (Haro et al., 2006). In contrast, NESDA and NESDO analyses reported that older age was associated with higher persistence of MDD (Schaakxs et al., 2018). However, these studies differ in key aspects, including a wider age range (18–88 years) with a substantial proportion of older adults and a focus on MDD in clinical samples, whereas our analyses included anxiety, mood, and substance use disorders in the general population. Our findings on childhood trauma do align with those from NESDA, where it was related to higher 2-year persistence of anxiety and/or depressive disorders (Hovens et al., 2012). Two additional determinants, living in a city and near-daily drug use, were also associated with higher persistence. Although direct comparisons are limited as prior studies have primarily focused on prevalence rather than persistence, these findings are consistent with evidence linking urban environments to higher depression prevalence and cannabis use frequency to greater psychiatric symptom burden, although the latter is based only on cross-sectional evidence (Xu, Miao, Turner, & DeRubeis, 2023; Yang et al., 2024). Sex was not associated with persistence of any mental disorder, consistent with previous longitudinal studies (Mackenzie et al., 201 4; Penninx et al., 2011). This highlights that prognostic risk factors do not necessarily mirror etiological risk factors, as female and male sex are well-established risk factors for the onset of anxiety/mood disorders and of substance use disorders, respectively (Eaton et al., 2011).

Although these determinants were associated with persistence, there was no consistent evidence that they explained the higher persistence observed in NEMESIS-3. The prevalence of younger age and living in a city among individuals with a 12-month mental disorder was modestly higher in NEMESIS-3 than in NEMESIS-2 (~20%), whereas no significant differences were observed for childhood trauma or near-daily drug use. Furthermore, the strength of the associations between these determinants and persistence did not differ significantly between studies, as confirmed by the nonsignificant multiplicative and additive interactions. Taken together, these findings suggest that changes in the prevalence or relative impact of these determinants are unlikely to explain the observed increase in persistence over time.

This study strengthens the existing literature by assessing 3-year persistence of DSM-5 anxiety, mood, and substance use disorders in a representative Dutch population using a structured diagnostic interview. It uniquely utilizes two comparable cohorts and clinically validated CIDI 3.0 (DSM-IV criteria) to examine trends in the 3-year persistence of common mental disorders (Haro et al., 2006). Moreover, it is the first study to apply a broad definition to the course of substance use disorders, whereas prior research has focused almost exclusively on narrow persistence with a focus on course of the index disorder only (Fenton et al., 2011; Mackenzie et al., 201 4). This broader approach provides insight into how individuals with substance use disorders may transition to other common mental disorders over time. Importantly, this is also the first study to investigate whether persistence itself has changed over time – an essential step toward a deeper understanding of trends in common mental disorders and the development of more tailored interventions.

Nevertheless, several limitations should be acknowledged. Although the CIDI 3.0 assessed DSM-IV common mental disorders with good validity (Haro et al., 2006), the validity and reliability of our slightly modified CIDI 3.0 for assessing DSM-5 diagnoses have not been formally evaluated (Ten Have et al., 2023). In addition, the sample size did not allow analyses of persistence by specific disorder subtypes, such as specific anxiety or substance use disorders. However, frequent and comparable transitions between anxiety disorder subtypes in recurrent anxiety suggest diagnostic instability and a shared underlying vulnerability, supporting a dimensional classification of anxiety disorders (Scholten et al., 2016). Similarly, the small sample sizes of the comorbidity groups limited our ability to detect significant changes in persistence trends between the two studies. Additionally, although the response rate in NEMESIS-3 was lower than in NEMESIS-2, this decline likely reflects a broader trend of decreasing participation in survey research, and weighting procedures were applied to minimize potential bias (Eggleston, 2024). Interpretation of these findings should be considered in the context of the Dutch healthcare system, which includes both primary care and specialized mental healthcare services, with strong primary care integration, universal insurance coverage, and relatively broad access to treatment compared with many other countries (Ravelli, 2006). However, access to mental healthcare varies across countries and is generally more limited in low- and middle-income settings (Patel et al., 2007; Saraceno et al., 2007), so direct comparisons of persistence trends in care use should be made with caution. Finally, although persistence was defined during the follow-up period, this may have included individuals whose disorder remitted shortly after baseline. Sensitivity analyses using a stricter 12-month recency definition for anxiety and mood disorders at follow-up yielded, as expected, slightly lower persistence rates. However, persistence rates were substantial and, most importantly, were consistently higher in NEMESIS-3 than in NEMESIS-2. Comparable analyses could not be performed for substance use disorders due to the lack of comparable 12-month follow-up data in NEMESIS-3.

This study shows that 3-year persistence of any anxiety, mood, and substance use disorder is high and has increased substantially over the past decade. Persistence was consistently higher when adopting a broad perspective, highlighting that many individuals continue to experience psychopathology even when the specific diagnosis changes over time. Younger age, living in a city, childhood trauma, and near-daily drug use were strongly associated with higher persistence, which emphasizes the importance of multiple factors in shaping persistence (defined as the presence of a disorder at any point during follow-up).

Our findings suggest that current mental health strategies may insufficiently address the long-term course of common mental disorders, alongside prevention and acute treatment efforts (Arango et al., 2018). To reduce the growing burden of these disorders, an integrated approach is needed that combines effective primary prevention, timely and effective treatment, and greater attention to long-term outcomes, including persistence and diagnostic transitions. Clinical practice and mental health policy therefore move beyond strictly disorder-specific models toward overarching strategies that acknowledge diagnostic transitions, comorbidity, and sustained recovery, particularly among younger adults, those living in cities, individuals with childhood trauma, and those with near-daily drug use. Greater emphasis on continuity of care and long-term monitoring may be key to reducing persistence and its associated societal and economic burden (Wijnen et al., 202 4).

## Supporting information

10.1017/S0033291726105467.sm001 Gkitakou et al. supplementary material Gkitakou et al. supplementary material

## Data Availability

The data on which this manuscript is based are not publicly available. However, data from NEMESIS are available upon request. The Dutch Ministry of Health financed the data, and the agreement is that these data can be used freely under certain restrictions and always under supervision of the Principal Investigator (PI) of the study. Thus, some access restrictions do apply to the data. The PI of the study is the second author of this article and can at all times be contacted to request data. At any time, researchers can contact the PI of NEMESIS and submit a research plan, describing its background, research questions, variables to be used in the analyses, and an outline of the analyses. If a request for data sharing is approved, a written agreement will be signed stating that the data will only be used for addressing the agreed research questions described and not for other purposes.

## Supplementary material

The supplementary material for this article can be found at http://doi.org/10.1017/S0033291726105467.

## Long descriptions

### Table 1. Long description

The table presents data for four groups from NEMESIS-3: Persons with any anxiety disorder (N equals 402), Persons with any mood disorder (N equals 371), Persons with any substance use disorder (N equals 290), and Persons with any mental disorder (N equals 828). All values are weighted percentages.

* Female: 61.8 for anxiety, 60.5 for mood, 32.9 for substance use, and 53.4 for any mental disorder.

* Young adults (18 to 34 years): 41.2 for anxiety, 41.0 for mood, 57.3 for substance use, and 45.7 for any mental disorder.

* Living without partner: 52.3 for anxiety, 53.8 for mood, 59.0 for substance use, and 53.2 for any mental disorder.

* No paid job: 41.0 for anxiety, 43.4 for mood, 29.9 for substance use, and 36.4 for any mental disorder.

* Education (Low): 26.6 for anxiety, 27.4 for mood, 21.2 for substance use, and 24.0 for any mental disorder.

* Education (Middle): 42.7 for anxiety, 44.9 for mood, 37.7 for substance use, and 42.7 for any mental disorder.

* Education (High): 30.6 for anxiety, 27.7 for mood, 41.1 for substance use, and 33.4 for any mental disorder.

* Living in a city: 64.9 for anxiety, 67.8 for mood, 66.7 for substance use, and 64.2 for any mental disorder.

Navigate back to Table 1..

### Table 2. Long description

The table presents data for 828 individuals from NEMESIS-3 with any mental disorder at T0. The broad perspective persistence for any mental disorder is 55.2 percent.

Disorder Categories (including those with other disorders):

* Any anxiety disorder: N equals 402. Narrow persistence 41.6 percent. Broad persistence 57.1 percent.

* Any mood disorder: N equals 371. Narrow persistence 48.0 percent. Broad persistence 63.2 percent.

* Any substance use disorder: N equals 290. Narrow persistence 40.3 percent. Broad persistence 55.1 percent.

Pure Disorders (excluding other disorders):

* Pure anxiety disorder: N equals 224. Narrow persistence 17.1 percent. Broad persistence 43.5 percent.

* Pure mood disorder: N equals 192. Narrow persistence 24.8 percent. Broad persistence 53.4 percent.

* Pure substance use disorder: N equals 206. Narrow persistence 32.4 percent. Broad persistence 50.9 percent.

Comorbid Disorders:

* Anxiety and mood disorder, no substance use disorder: N equals 122. Narrow persistence 66.1 percent. Broad persistence 75.8 percent.

* Anxiety and substance use disorder, no mood disorder: N equals 27. Narrow persistence 34.0 percent. Broad persistence 63.5 percent.

* Mood and substance use disorder, no anxiety disorder: N equals 28. Narrow persistence 41.3 percent. Broad persistence 67.0 percent.

* All three disorders: N equals 29. Narrow and broad persistence are both 62.5 percent.

Navigate back to Table 2..

### Table 3. Long description

The table is structured with 11 columns. The first column lists DSM-IV disorders at T0. Subsequent columns provide N at T0 for NEMESIS-2 and NEMESIS-3, followed by two main sections: Narrow Perspective (same diagnostic category) and Broad Perspective (any mental disorder). Each section includes percentages with 95 percent C I for both studies, p-values, and sex- and age-adjusted odds ratios (a O R).

Key data points include:

* Any mental disorder: NEMESIS-2 broad persistence was 42.7 percent versus 57.9 percent in NE MESIS-3 (p < 0.01, a O R 1.81).

* Any anxiety disorder: Narrow persistence increased from 24.4 percent to 40.1 percent (p < 0.01).

* Any mood disorder: Narrow persistence increased from 33.5 percent to 48.8 percent (p < 0.01).

* Pure anxiety disorder: Narrow persistence increased from 8.0 percent to 18.3 percent (p = 0.02).

* Pure mood disorder: Narrow persistence increased from 13.7 percent to 26.0 percent (p < 0.01).

* Pure substance use disorder: Narrow persistence increased from 17.5 percent to 36.5 percent (p < 0.01).

* Comorbid disorders: Persistence rates are generally higher but show less statistical significance between studies. For example, ‘All three disorders’ shows narrow persistence of 88.6 percent in NEMESIS-2 and 80.6 percent in NE MESIS-3 (p = 0.53).

Navigate back to Table 3..

### Table 4. Long description

The table lists determinants for mental disorder persistence across three columns: Determinants, Unadjusted model O R (95 percent C I), and Adjusted model a O R (95 percent C I).

Key findings with significant odds ratios (bolded) in both models include:

* Young adult (18-34 years): O R 1.92 (1.53, 2.42); a O R 1.88 (1.35, 2.61).

* Living in a city: O R 1.35 (1.03, 1.76); a O R 1.40 (1.05, 1.87).

* Childhood trauma: O R 1.44 (1.11, 1.86); a O R 1.48 (1.13, 1.94).

* Near-daily drug use: O R 1.73 (1.22, 2.43); a O R 1.71 (1.15, 2.56).

Determinants significant only in the unadjusted model include:

* Living without partner: O R 1.54 (1.22, 1.93).

* No paid job: O R 1.34 (1.04, 1.73).

* Low income: O R 1.82 (1.27, 2.61).

* Middle income: O R 1.49 (1.07, 2.08).

* Smoking: O R 1.40 (1.09, 1.81).

Non-significant determinants include Female gender, Education levels (Low and Middle), Negative life events, Physical inactivity, Chronic physical conditions, and Alcohol use frequency (Non-daily and Near-daily drinking).

Navigate back to Table 4..
