# Supplementary material for: Are common mental disorders more persistent over time? Insights from the Netherlands Mental Health Survey and Incident Studies (NEMESIS)
Source: Psychol Med. 2026 Sep 3;56:e258. doi: 10.1017/S0033291726105467 (PMC13735845; doi:10.1017/S0033291726105467)
Supplement: Supplementary file 1 [file S0033291726105467sup001.docx]

S. Table 1: Determinants of three-year persistence of any DSM-IV mental disorder (broad definition) among respondents with any 12-month disorder at baseline in each study (NEMESIS-2 and NEMESIS-3; 18-64 years), differences in the strength of these determinants were tested using multiplicative and additive interaction, in weighted odds ratios (OR)

| Any mental disorder (broad perspective) | **NEMESIS-2 (2007-2012)** | | **NEMESIS-3 (2019-2024)** | | | **Multiplicative interaction** | **Additive interaction** |
| --- | --- | --- | --- | --- | --- | --- | --- |
|  | % | OR (95% CI) | % | OR (95% CI) | OR (95% CI) | | RD (95% CI) |
| **Determinants** |  | N=703 |  | N=691 |  | |  |
| Female | 52.2 | 0.96 (0.66, 1.39) | 52.0 | 1.16 (0.82, 1.65) | 1.22 (0.72, 2.04) | | 0.05 (-0.08, 0.17) |
| Young adults (18-34 years) | 42.6 | 1.95 (1.39, 2.72) | 51.5 | 1.77 (1.27, 2.44) | 0.91 (0.57, 1.45) | | -0.02 (-0.14, 0.09) |
| Living without partner | 54.6 | 1.90 (1.32, 2.73) | 56.9 | 1.25 (0.90, 1.73) | 0.66 (0.39, 1.10) | | -0.10 (-0.23, 0.02) |
| No paid job | 31.1 | 1.19 (0.78, 1.83) | 33.8 | 1.46 (1.05, 2.01) | 1.22 (0.71, 2.10) | | 0.05 (-0.09, 0.18) |
| Education (ref= high) |  |  |  |  |  | |  |
| Low | 34.1 | 1.29 (0.82, 2.02) | 22.5 | 1.27 (0.90, 1.78) | 0.98 (0.57, 1.71) | | 0.00 (-0.14, 0.13) |
| Middle | 42.2 | 1.03 (0.72, 1.47) | 44.5 | 1.15 (0.81, 1.62) | 1.11 (0.73, 1.70) | | 0.03 (-0.08, 0.13) |
| Living in a city | 52.7 | 1.13 (0.76, 1.69) | 63.6 | 1.43 (1.02, 2.01) | 1.27 (0.75, 2.14) | | 0.06 (-0.07, 0.19) |
| Income (ref=high) |  |  |  |  |  | |  |
| Low | 35.2 | 1.97 (1.06, 3.69) | 37.4 | 1.85 (1.15, 2.96) | 0.94 (0.41, 2.12) | | -0.01 (-0.20, 0.18) |
| Middle | 49.5 | 1.75 (1.09, 2.81) | 44.0 | 1.46 (0.89, 2.41) | 0.83 (0.40, 1.71) | | -0.04 (-0.20, 0.13) |
| Negative life event | 69.2 | 1.25 (0.79, 1.97) | 66.6 | 1.08 (0.75, 1.56) | 0.87 (0.48, 1.56) | | -0.03 (-0.18, 0.11) |
| Childhood trauma | 46.1 | 1.51 (1.03, 2.21) | 43.7 | 1.45 (1.04, 2.03) | 0.96 (0.59, 1.57) | | -0.01 (-0.13, 0.11) |
| Smoking | 45.7 | 1.66 (1.14, 2.41) | 31.9 | 1.48 (1.02, 2.14) | 0.89 (0.53, 1.50) | | -0.03 (-0.15, 0.10) |
| Not physically active | 60.6 | 0.99 (0.68, 1.45) | 52.3 | 1.45 (1.08, 1.94) | 1.46 (0.92, 2.33) | | 0.09 (-0.02, 0.21) |
| ≥1 chronic physical conditions | 39.1 | 0.90 (0.64, 1.26) | 38.3 | 0.96 (0.75, 1.23) | 1.07 (0.70, 1.65) | | 0.02 (-0.09, 0.12) |
| Alcohol use frequency (ref= non-drinking) |  |  |  |  |  | |  |
| Non-daily drinking | 63.2 | 0.87 (0.53, 1.44) | 67.4 | 0.76 (0.52, 1.11) | 0.88 (0.49, 1.57) | | -0.03 (-0.17, 0.11) |
| Near-daily drinking | 21.4 | 0.78 (0.44, 1.37) | 11.2 | 0.96 (0.54, 1.72) | 1.24 (0.59, 2.61) | | 0.05 (-0.13, 0.23) |
| Near-daily drug use | 11.5 | 1.69 (0.94, 3.03) | 9.9 | 1.94 (1.13, 3.34) | 1.15 (0.48, 2.75) | | 0.02 (-0.18, 0.22) |

OR: odds ratio; CI: confidence intervals; RD: risk difference; ref: reference.

Statistically significant (p-value<0.05) differences in unadjusted odds ratios (ORs) for multiplicative interactions and risk differences (RDs) for additive interactions are indicated in **bold**.

Data were weighted using post-stratification to ensure generalization to Dutch population.
